# Towards agreement amongst parents, teachers and children on perceived psychopathology in children in a Kenyan socio-cultural context: a cross-sectional study

**DOI:** 10.1186/s12888-024-05679-2

**Published:** 2024-04-05

**Authors:** Victoria Mutiso, David M. Ndetei, Christine Musyimi, Jenelle Shanley, Monica Swahn, Kamaldeep Bhui

**Affiliations:** 1 Africa Mental Health Research and Training Foundation, Nairobi, Kenya; 2 Department of Psychiatry, University of Nairobi, Nairobi, Kenya; 3 World Psychiatric Association Collaborating Centre for Research and Training, Nairobi, Kenya; 4 School of Graduate Psychology, Pacific University, Hillsboro, USA; 5 Department of Health Promotion and Physical Education, Wellstar College of Health & Human Services, Kennesaw State University, Kennesaw, GA USA; 6 Department of Psychiatry, University of Oxford, England, UK

**Keywords:** Psychopathology, TRIAD, Children, Parents, Teachers

## Abstract

**Background:**

Our objective was to determine levels of agreement between parents, teachers and children on mental symptoms in the children. Teachers, children and parents constitute the TRIAD in the perception of psychopathology in children. Analyzing the perceptions of psychopathology from the perspectives of parents, teachers, and children is essential for a comprehensive understanding of a child’s mental health.

**Methods:**

We identified 195 participants across ten randomly sampled primary schools in South East Kenya. Potential participants were randomly selected and a sampling interval calculated to determine the study participants. The children (Class 5–8; aged 11–14) completed the Youth Self-Report (YSR) scale, the parents the Child Behavior Check List (CBCL) on their children and the teachers completed the Teachers Rating Form (TRF) on the children. Only parents and teachers who gave consent as well as children who gave assent were included in the study. Analysis was conducted using Stata 14.1 and Pearson correlation coefficients used to calculate the correlations between CBCL, YSR and TRF.

**Results:**

The children agreed least with the parents and more with the teachers. There was a greater agreement between the children and their teachers in 5 (2 internalizing disorders and 3 externalizing disorders) out of the 8 conditions. Children and parents agreed only on somatic disorders and conduct disorders. YSR mean scores were significantly lower than those for CBCL for all problem scales. Mean scores of TRF and YSR were comparable in the majority of the problems measured.

**Conclusion:**

We suggest broad-based psychoeducation to include children, parents/guardians and teachers to enhance shared awareness of psychopathology and uptake of treatment and for the consideration of an integrated mental health system.

## Introduction

A standard practice in the management of disorders in children is to seek corroboration on information from different sources, including children and those who spend the most time with children such as parents and teachers [1]. Home and school environments are not identical and may inhibit, allow or facilitate the manifestation of different types of behavior or give different manifestations of the same underlying pathology [2]. For instance, school environments tend to be highly structured with rules and regulations that apply equally to all children while family environments are less structured and allow more emotional involvement [3]. There could be variations between children, parents and teachers on how they perceive the same conditions: Children may not be objective in rating themselves on such conditions such as hyperactivity, inattention and oppositional behavior, but teachers and parents/caregivers are likely to be more objective; children themselves and their parents may be more objective than teachers on internalizing problems which are more observable in a one to one basis in a family environment [4, 5]. Mothers and fathers do not always agree on all items of Child Behavior Check List (CBCL), even though they are rating the same children [6]. Furthermore, not all attributes of a child may be easily discernable to either teachers, parents or clinicians tasked with the management of the disorders in children– for example, the sympathy of the children for one another [7], which is more likely to be exhibited directly to other children– at home or school. A more recent meta-analysis of 169 studies [8] found small to moderate agreement between parents and youths (0.33–0.40) and between parents and teachers (0.18–0.35). The mean level disagreement was related to youth characteristics, parent characteristics, assessment context and scale measured with BERS-2 possessing moderate to a high cross-informer agreement with co-efficient ranging from 0.50 to 0.63 [9].

Understanding psychopathology perception in adolescents is very crucial because this stage of life is marked by significant changes both physically, emotionally and socially. During this stage of life, stigma surrounding mental health, perpetuated by families, friends and societal norms, can significantly impact adolescents’ perceptions and experience of psychopathology [10]. They may be reluctant to acknowledge or seek help for mental health issues because they feel embarrassed and fear being judged by their peers for admitting to struggling with mental health challenges [11]. Additionally, they may have concerns over confidentiality and trust in their relationship with health care professionals, as fears of judgment or breaches of privacy may discourage them from fully disclosing their struggles and seeking the support [12].

As far as we could establish, there is no Kenyan data on the perception of psychopathology amongst school going children, their teachers and parents. Such information would inform and justify integrated approach to the management of psychopathology in children. The overall aim of this study was to provide local Kenyan information and to fill the gap on how parents, teachers and children themselves perceived psychopathology in the children. The specific aims were: [1] To provide data on the levels of agreement or disagreement on the key TRIAD of CBCL by parents on their children, Youth Self-Report (YSR) by children on themselves and Teachers Rating Form (TRF) by the teachers on the same children in a Kenyan socio-cultural setting; [2] To generate Kenyan evidence for the feasibility of a three-pronged approach to the management of psychopathology in school going children.

## Methods

### Participants

This was a cross sectional study. The participants (non-clinical) were drawn from ten schools in Machakos Sub-Country in South East Kenya. To facilitate effective supervision of schools by the school supervisors, the schools in Machakos sub-county were divided into several groups; each group was referred to by the Ministry of Education (MoE) as a cluster. We randomly chose ten clusters and then randomly selected one school per cluster to meet our predetermined sample. This sampling procedure had been used successfully in another study but excluded ten schools used for this study [13]. Children were recruited from their respective classes (Class 5–8; aged 11–14). In Kenya, the primary school age range spans from 6 to 14 years hence the sample was drawn from primary schools. Occasionally, you will find pupils exceeding the age of 14 within the primary school, a reflection of individual circumstances tied to the age at which they first entered school. This age range, often referred to as early adolescence, encompasses a crucial period of cognitive, emotional, and social development, which may influence perceptions of psychopathology. For each class, in the ten schools, potential participants were randomly selected. Based on the average number of students per class, a sampling interval was calculated using this formula: $\varvec{k}=\frac{\varvec{N}}{\varvec{n}}$ where; k = sampling interval, N = population of children in a class, and n = number of children to be sampled [14]. Every k^th^ individual who was sampled was recruited into the study until the minimum sample was met. Whenever a child was selected for the study and the parent was not available/ to give consent, the next child whose parent was available and consented to be part of the study was selected. The inclusion criteria included being in a primary school, informed assent by the students and consent by the parents/guardians with a right to withdraw any time without any loss of benefits and no obvious cognitive deficits on the part of the child. The exclusion criteria included not being a student in the primary school, no informed assent/consent and no severe cognitive deficits. All the approached students met the inclusion criteria.

### The research assistants (RAs)

We undertook a 2-day training for 20 RAs, 2 for each school on how to administer our research instruments i.e. YSR, CBCL and ASR by reading the questions to a parent up to 3 times without any elaboration and then recording the answer. If they still did not understand by the third time, the RAs were trained to skip that question. The training included reading through all the questions in a group and administering the questions to one another in the group until there was uniformity in which all the RAs read each of the questions.

### The instruments

Three psychometric instruments– CBCL, YSR and TRF– were used. These tools were chosen because they are widely recognized and established tools for assessing psychopathological symptoms in children. The tools have demonstrated high levels of validity and reliability in various studies across diverse populations [15–17]. The process of adaptation of the instruments (to ensure original meaning was retained; back and forth translation from English to Swahili and the local dialect, Kamba, piloting and adoption) has already been reported for CBCL and YSR [13, 18]. We repeated the same process for TRF. This translation was done in consultation with the authors of Achenbach System of Empirically Based Assessment (ASEBA) to satisfy them that the back-translated versions reflected the original meaning and concepts behind the original questionnaires developed by them. We, therefore, used back translations that were approved by the authors of ASEBA.

1. *The Child Behavior Check List (CBCL)* measures internalizing and externalizing behavior problems in children as perceived by the parents [17]. The questionnaire contains 113 items which are rated on a 3-point Likert scale (0 = ‘not true’, 1 = ‘sometimes true’ or ‘somewhat true’, 2 = ‘often true’ or ‘very true’). This instrument has good psychometric properties with high levels of internal consistency (Cronbach’s alpha range of 0.72 to 0.91 for DSM-IV oriented scales) and test-retest reliability of *r* =.97 [15]).
2. *The Youth Self-Report (YSR)* is a self-report measure completed by children aged 11–18 to assess emotional and behavioral problems. It has demonstrated excellent consistency during comparisons across different and multicultural societies [16] and has good psychometric properties with high levels of internal consistency (Cronbach’s alpha range of 0.67 to 0.82 for DSM-IV oriented scales), as well as high test-retest reliability (*r* =.88) [19].
3. *The Teachers Rating Form (TRF)* is completed by teachers and other school staff to assess problem behavior, academic performance and adaptive functioning of the children. It has 113 items and takes averagely 15 min to complete and 10 min to score. It has a good internal consistency of between 0.73 and 0.94 on DSM-IV scales and test-retest reliability of *r* =.72 to 0.95 [20]. The cutoff point for the scores of this instrument is done using a software developed by ASEBA [21].

### Data collection

We approached the ten primary schools and explained the nature of the study to the head teachers for their permission, followed by the school boards and the Parents Teachers Association (PTA) for their informed permission to undertake the study. The parents were approached during the regular parents and teachers school meetings, during which the nature of the study was explained and they were given time to ask for any clarifications. We then asked for their consent and consent for their children to participate in the study. The children were given information about the nature of the study and asked for their assent. Only parents, their children and class teachers for whom we had consent and assent were included in the study. For all the children included in the study we approached their teachers (a teacher for several studies in their classroom) and parents to complete their respective tools. Each class teacher completed the TRF for all the children in their class, but only included for analysis only those children for who we had data by the children and by the parents. The RAs administered the questions after checking the validity of all the consents and assents.

### Data management and analysis

The data from the YSR, CBCL and TRF were double entered and scored by the Assessment Data Manager (ADM) software version 9.1, a tool developed by the ASEBA team [22]. The ADM scores each assessment and produces a summation of all problem items and ratings for the DSM-IV Oriented Scale separately for boys and girls and standardized T-scores. Both raw and scored datasets, as well as the socio-demographic data, were converted into SPSS through A2S (a one-way utility designed to process data from Assessment Data Manager (ADM) or Ratings-to-Scores (RTS) into SPSS already scored.

The analysis was conducted using Stata 14.0. The level of agreement between raters and across scales was examined. Descriptive statistics were used to examine the general distribution of data of the participants and YSR/CBCL, YSR/TRF and CBCL/TRF scales. We then calculated Pearson correlation coefficients and intra-class correlation coefficients (ICC) between YSR/CBCL, YSR/TRF and CBCL/TRF problem severity difference scores. We calculated Cohen’s kappa (κ) statistic to assess chance agreement. Analysis of variance (ANOVA) was used to examine discrepancies between YSR–CBCL and TRF scores. To determine the cut-off points for clinical and non-clinical level scores we used the same cut-off points used by ASEBA and which have been validated in several cross-cultural studies [21].

## Results

The final sample for which we had complete matched data was of 195 children (*n* = 104, 53.3% females, mean age = 14.3, *SD* = 2.2, range = 11–19 years; *n* = 91, 46.7%, mean age = 14.2, SD = 2.2 range = 11–19), 154 mothers (mean age = 38.8 years, *SD* = 6.0, range 25–61 years) and 41 fathers (mean age = 41.7 years, *SD* = 8.3, range 20–61 years). Concerning teacher informants, the majority (59.3%) were classroom teachers and 40.7% were males (we were unable to get the age of teachers since TRF does not capture details of teachers ages at the same time the exact number of teachers who gave information about their pupils could not be ascertained since one teacher would rate multiple students).

Table 1 presents the results of correlations between CBCL, YSR and TRF.

*Correlations between parent-reported scores (CBCL) and self-reports (YSR):* Significant positive correlation was found between YSR and CBCL on somatic disorders and conduct problems.

*Correlations between teacher reported scores (TRF) and self-reports (YSR):* Significant positive correlation was found between total problems, affective disorders, anxiety disorders, somatic disorders and oppositional disorders.

*Correlations between teacher reported scores (TRF) and parent report (CBCL):* There were significant positive correlations between somatic and ADHD problems.

**Table 1:** Pearson’s correlations between CBCL, YSR and TRF A = Between children perception (YSR) and parents perception (CBCL)

**Pearson’s Correlations**	**CBCL**								
**A = Between children perception (YSR) and parents perception (CBCL)**									
**YSR**	1	2	3	4	5	6	7	8	9
1. Internalizing	0.04								
2. Externalizing	-0.004	0.081							
3. Total Problems	0.011	0.087	0.038						
4. Affective disorders	0.066	0.147^*^	0.095	0.094					
5. Anxiety disorders	0.032	0.054	0.045	0.042	-0.034				
6. Somatic disorders	0.027	0.046	0.038	0.031	-0.058	0.187^*^			
7. ADHD	0.057	0.155^*^	0.071	0.032	0.062	0.06	0.07		
8. Oppositional disorders	0.068	0.129	0.116	0.11	0.031	0.036	0.019	0.061	
9. Conduct disorders	0.02	0.11	0.053	0.048	0.04	-0.005	0.016	0.066	0.195^**^
**Pearson’s Correlations**	**TRF**								
**B = Between children (YSR) and teachers (CBCL)**									
**YSR**	1	2	3	4	5	6	7	8	9
1. Internalizing	0.116								
2. Externalizing	0.042	0.123							
3. Total Problems	0.119	0.221^**^	0.182^*^						
4. Affective disorders	0.151^*^	0.249^**^	0.217^**^	0.156^*^					
5. Anxiety disorders	0.149^*^	0.173^*^	0.158^*^	0.136	0.177^*^				
6. Somatic disorders	0.074	0.206^**^	0.152^*^	0.016	0.09	0.145^*^			
7. ADHD	0.145^*^	0.194^**^	0.181^*^	0.133	0.142^*^	0.009	0.13		
8. Oppositional disorders	0.229^**^	0.242^**^	0.279^**^	0.322^**^	0.224^**^	0.160^*^	0.215^**^	0.306^**^	
9. Conduct disorders	-0.019	0.062	0.071	0.062	0.022	-0.032	0.092	0.115	0.1
**Pearson’s Correlations**	**TRF**								
**C = Between parents (CBC) and teachers (CBCL)**									
**CBCL**	1	2	3	4	5	6	7	8	9
1. Internalizing	0.019								
2. Externalizing	-0.014	0.073							
3. Total Problems	0.012	0.128	0.055						
4. Affective disorders	-0.069	-0.027	-0.068	-0.058					
5. Anxiety disorders	-0.032	-0.003	-0.019	-0.006	-0.045				
6. Somatic disorders	-0.041	-0.017	-0.064	-0.066	-0.034	0.148^*^			
7. ADHD	0.033	0.112	0.108	0.095	0.048	0.038	0.209^**^		
8. Oppositional disorders	-0.047	-0.012	0.001	-0.037	-0.07	-0.052	0.018	-0.016	
9. Conduct disorders	-0.083	0.021	-0.007	-0.009	-0.06	-0.061	0.068	0.056	0.002

**. Correlation is significant at the 0.01 level (2-tailed). *. Correlation is significant at the 0.05 level (2-tailed)

Tables 2 and 3 present the results of the comparison of syndrome scales between self-reported, parent-reported and teacher-reported scores and assess agreement between the pairs of raters and measures.

There were significant differences in reported scores between the children, parents and teachers in all the scores apart from conduct disorders where they were in agreement. Using LSD for Post hoc tests for pair-wise comparison, significant differences were found between Parent-Teacher and Parent-Child reporting for internalizing syndromes, Child and teacher for externalizing syndromes, Parent and child for total problems, Parent-Child and Parent-Teacher for affective disorders, Parent-Child and Child-Teacher for anxiety disorders, Child-Teacher and Parent-Teacher for somatic disorders, Child-Teacher for ADHD, Parent-Child and Child-Teacher for oppositional disorders. There were no significant differences in the scores of conduct disorders.

### Agreement across children, teachers and parents

The level of agreement between child and parent was low on most of the syndrome scores apart from externalizing problems k = 0.147; *P* =.0489 and conduct problems k = 0.190; *P* =.042. The level of agreement was low in all of the syndrome scores except conduct problems k = 0.248; *p* =.0003 between the child and the teacher. The level of agreement between the teacher and parent was low in most of the syndrome scores except for somatic k = 0.155, *P* =.0102 and ADHD problems k = 0.563, *P* <.001. The ICC in all the syndrome scales was below 0.7 which is below the recommendation by Cohen.

*NOTE:* The general recommendation by Cohen was that reliability be above 0.7, which means 70% of the observed variance is “real” variance. But it depends on what you’re willing to accept, or what the research literature suggests is typical/necessary.

**Table 2:** Level of agreement between the pairs of raters and Measures the inter-rater reliability of a scale mean (TRF, CBCL and YSR)-Intra-class correlation coefficient (ICC)

Syndrome	Child and Parent		Child and Teacher		Parent and Teacher		ICC	95% C.I
	Kappa (S.E)	Approximate Significance	Kappa (S.E)	Approximate Significance	Kappa (S.E)	Approximate Significance		
Internalizing	0.070 (0.07)	0.3079	0.021 (0.07)	0.7714	0.005 (0.07)	0.941	0.071	-0.18 to 0.27
Externalizing	0.147 (0.10)	**0.0489**	0.095 (0.09)	0.1926	0.024 (0.09)	0.743	0.186	-0.04 to 0.37
Total Problems	-0.007 (0.07)	0.9254	0.064 (0.08)	0.3793	0.060 (0.08)	0.405	0.083	-0.17 to 0.29
Affective disorders	0.031 (0.08)	0.6676	0.179 (0.11)	**0.0098**	0.040 (0.11)	0.523	0.168	-0.05 to 0.35
Anxiety disorders	0.074 (0.09)	0.3105	0.257 (0.12)	**0.0004**	0.042 (0.12)	0.566	0.303	0.11 to 0.46
Somatic disorders	0.103 (0.08)	0.1682	0.107 (0.08)	0.0844	0.155 (0.08)	**0.010**	0.28	0.09 to 0.44
ADHD	-0.009 (0.01)	0.8795	-0.008 (0.01)	0.8978	0.563 (0.01)	**0.000**	0.488	0.35 to 0.60
Oppositional disorders	-0.008 (0.01)	0.8958	-0.013 (0.01)	0.8555	-0.007 (0.01)	0.916	-0.038	-0.33 to 0.20
Conduct disorders	0.190 (0.13)	**0.0042**	0.248 (0.13)	**0.0003**	-0.031 (0.13)	0.675	0.302	0.11 to 0.46

Note: The ICC was computed with 3 raters across 189 rates

**Table 3:** Differences between YSR, CBCL and TRF scores

Syndrome	YSR	CBCL	TRF	F	P
Internalizing	58.1 ± 10.1	60.9 ± 10.4	58.8 ± 9.9	3.57	**0.0288**
Externalizing	51.3 ± 10.3	52.8 ± 9.3	54.6 ± 8.6	5.79	**0.0032**
Total Problems	53.9 ± 11.4	57.0 ± 10.9	55.3 ± 9.2	3.68	**0.0257**
Affective disorders	58.3 ± 7.9	60.7 ± 8.1	56.9 ± 7.7	10.79	**0.0000**
Anxiety disorders	55.5 ± 6.4	58.1 ± 6.9	58.2 ± 7.6	8.61	**0.0002**
Somatic disorders	61.0 ± 10.1	61.6 ± 10.1	54.5 ± 8.0	32.46	**0.0000**
ADHD	53.0 ± 4.6	54.0 ± 5.3	54.3 ± 5.4	3.36	**0.0354**
Oppositional disorders	52.3 ± 4.3	54.5 ± 5.5	53.6 ± 5.3	9.58	**0.0001**
Conduct disorders	56.8 ± 8.3	56.3 ± 6.7	55.6 ± 6.4	1.26	0.2855

Note: F-Values are based on ANOVA by tool of assessment

### Disagreement across children, teachers and parents

**Fig. 1:**
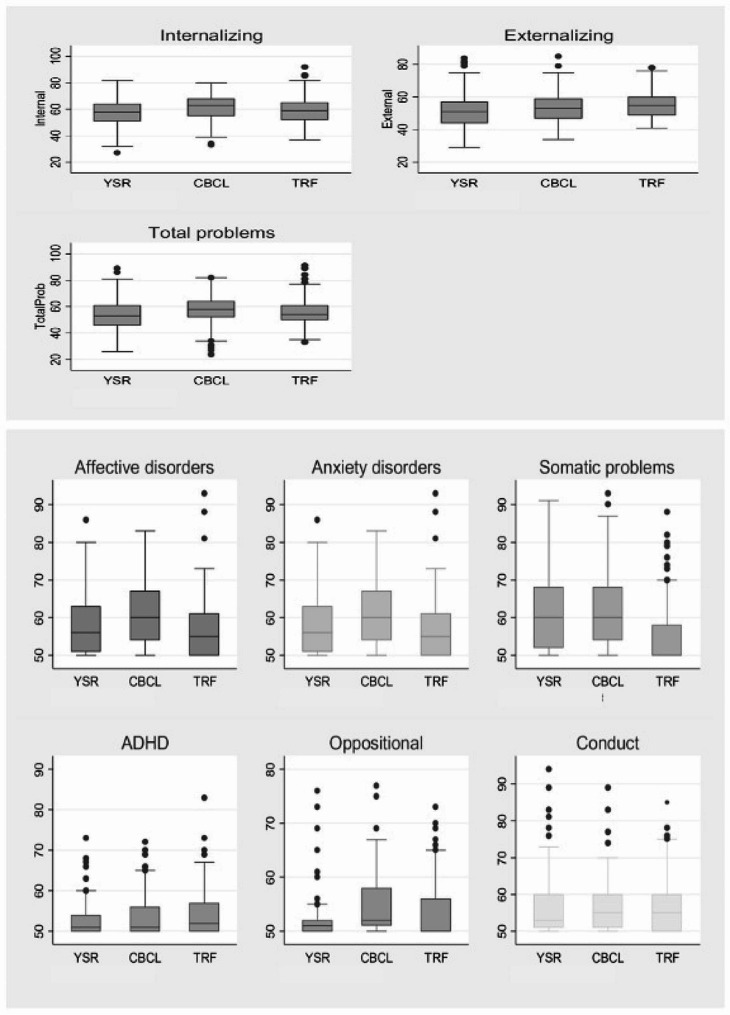
Graphical representation of different syndromes by different raters The dots represent outliers with YSR reporting more among the syndromes except for Affective disorder, Somatic problem, Total problems and Internalizing problem; mean disagreement scores for the syndromes for children, parents and teachers differ; Children disagreed more with the teachers and parents on all the syndromes except for externalizing problems and also affective disorder, anxiety disorder, ADHD, Oppositional, Conduct; Parents disagreed more with children and teachers on affective disorder, anxiety disorder, oppositional; teachers disagreed more with parents and children on externalizing problem, ADHD, Oppositional and Conduct

These disagreements in the majority of cases were confirmed when the means scores by parents, teachers and children were computed except for conduct disorders and reconfirmed on tests of inter-rater and intra-class correlation coefficient. These trends are graphically illustrated in Fig. 1.

## Discussion

We report here the first Kenyan study that used the TRIAD of CBCL, YSR and TRF on 195 children attending school in a Kenyan setting. Our findings are in agreement with most studies reviewed in the Introduction to the effect there is little agreement between CBCL, YSR and TRF. There was a greater agreement between the children and their teachers in 5 (2 internalizing disorders (affective and anxiety) and 3 externalizing disorders (somatic, ADHD and oppositional) out of the 8 conditions. Our results suggest that children and parents agreed only on somatic disorders and conduct disorders.

These findings can be understood in the Kenyan context. Somatic disorders are mainly physical symptoms, often perceived to suggest physical conditions, and not normally perceived by the parents of the children as suggestive of mental disorders [23]. Conduct disorders include substance use which the parents may have noticed and therefore agreement between parents and children even though they may not have talked about them. In the Kenyan context, in the day-school system (not boarding schools) children spend more time with teachers (8am– 3.45pm, Monday to Friday). Substantial time is spent moving from home to school (normally walking) to be in class by 8am and equally the same time to go back home. Most of the evening is taken up with homework. All of these contribute to the less time (in quality and quantity) the children spend with their parents/guardians.

On the other hand, parents and children who spend less time together than with the teachers agreed on only 2 conditions– one internalizing (anxiety) and the other externalizing (ADHD). The agreement on these 2 conditions is not surprising: anxiety is likely to result in school phobia and failure to go to school, which would be obvious to both parents and the teachers; ADHD will lead to disruptive behavior in the structured environment found in schools. The teachers immediately share this with parents all of whom may view as requiring a disciplinary approach which needs their connected effort.

Our study was on a non-clinical population. This is unlike a clinical population in which parents would have a greater role in initiating referral and therefore expected to have noticed abnormalities. This is yet another reason why parents and children agree only on externalizing processes and conduct disorders.

Our findings compare and contrast with other studies. A population study [24] similar to ours, found the mean for YSR to be higher than the mean for CBCL. We, on the other hand, found the opposite i.e. CBCL mean score higher than YSR, CBCL also higher than TRF and more similarities between YSR and TRF. It is possible that internalizing factors and some externalizing factors were highly noticeable by the parents and therefore contributed to this observation. The agreement between YSR and TRF could be a reflection of the fact that children spent more time with teachers than with parents.

Apart from time spent with children, there are other plausible and overlapping explanations for these overall disagreements between parents and teachers and parents and children: (i) Home and school environments are not the same and may lead to different expressions of the same disorder as was observed by Des Los Beyes [25], leading to different perceptions by parents and teachers; (ii) Children, parents and teachers have different perceptions on what constitutes abnormality in the behavior of the children, except when the behaviors on the part of the children are dramatic enough to catch the attention of all such as refusal to go to school on account of school phobia, truancy, etc. These may be viewed by both teachers and parents as requiring a disciplinary approach while the children perceive themselves as helpless victims as may occur in ADHD. As already explained, somatic symptoms in the Kenyan context in school-going children would be regarded by most as physical disorders [23] and not stigmatized as they would be perceived as genuine and therefore attract the attention of all players including the children. Although clinicians and researchers may be more comfortable with more agreement between different informants as pointed out in the introduction, these different disagreements are clinically important and useful as they provide a much wider scope to the understanding of the problems from different perspectives and factors thus forming a basis to work towards convergent perspectives. Our findings, therefore, suggest a family or school or combined oriented psychoeducation on mental health symptoms towards a common perspective of the problem. It calls for a multi-disciplinary team approach where the teachers, parents/guardians and service providers and the children themselves are key stakeholders. This kind of approach is no less desirable in low-resourced countries than it is in high-resourced countries. There is a need to start somewhere with the resources already available to mitigate today’s needs but there is also the need to be innovative [13]. However, part of the innovation is the ability to address both covert and overt power structures between children, teachers and parents. In the Kenyan socio-cultural context, there is a common uniting factor in these structures and that is the shared critical importance attached to the education of children as the best investment for the child, the family, the respect for the teachers and the benefits to the community. These considerations transcend power structures when it comes to matters of education for children. Indeed, we have demonstrated that it is possible to establish such a dialogue [26].

Bringing together the perceptions of teachers, parents and the children to find a common understanding on psychopathology in the child will enhance an integrated approach at clinical level. More importantly, this same approach can find a community health application that will have a critical reach in enhancing all round awareness in the key players. To achieve this, practical steps can be implemented to foster shared awareness within the community. Initiatives such as community-based awareness programs and school-based mental health interventions serve as valuable tools in this endeavor. In addition to broader community initiatives, targeted measures can be implemented to empower educators. Training programs for teachers on recognizing early signs of psychopathology are crucial components of a comprehensive approach. Equipping teachers with the necessary knowledge and skills enables them to play a proactive role in identifying and addressing mental health concerns among students. Complementary to teacher training, community outreach initiatives serve as an important means to connect with families and individuals in various socio-cultural contexts. By extending mental health support beyond the classroom, these initiatives contribute to a more inclusive and accessible system, ensuring that the broader community is well-informed and engaged in the promotion of mental wellbeing. A major limitation of this study is the small sample size. Further mitigation against this limitation is that we only worked with participants who willingly came forward to participate in the study, and provided we had data on the same students from parents and teachers.

The strengths of this paper lie in our methodology where we asked all the participants the same questions in a standardized format by trained RAs to achieve the highest possible inter-and intra-rater reliability. Another strength is that we used intervention on populations who had not been exposed to any mental health awareness, psychoeducation or clinical intervention.

We have achieved the overall aim and the specific aims. In the process, we have demonstrated the feasibility of this kind of research in Kenya and laid the grounds for future research on the possible efficacy and effectiveness of this kind of approach at the clinical level. We have built the case for integrated mental health systems comprising children, parents and teachers for the management of childhood disorders in a Kenyan setting. We have contributed to the global database by demonstrating similarities between HIC and our Kenyan findings.

## Data Availability

The data for this study will be made available upon written request to the corresponding author detailing the specific parts of the data to be shared and the intended purpose.
